# Macrophage – tumor cell interaction beyond cytokines

**DOI:** 10.3389/fonc.2023.1078029

**Published:** 2023-02-23

**Authors:** Olga Kovaleva, Maxim Sorokin, Anastasija Egorova, Anatoly Petrenko, Ksenya Shelekhova, Alexei Gratchev

**Affiliations:** ^1^ Laboratory for Tumor Stromal Cell Biology, Institute of Carcinogenesis, Nikolaj Nikolajevich (N.N.) Blokhin National Medical Research Center of Oncology, Moscow, Russia; ^2^ Department of Pathology, Clinical Research and Practical Center for Specialized Oncological Care, St. Petersburg, Russia; ^3^ Pathology Department, SPb Medico-Social Institute, St. Petersburg, Russia

**Keywords:** macrophage, exosome, cancer, miRNA, lncRNA

## Abstract

Tumor cells communication with tumor associated macrophages is a highly important factor of tumor malignant potential development. For a long time, studies of this interaction were focused on a cytokine- and other soluble factors -mediated processes. Discovery of exosomes and regulatory RNAs as their cargo opened a broad field of research. Non-coding RNAs (ncRNAs) were demonstrated to contribute significantly to the development of macrophage phenotype, not only by regulating expression of certain genes, but also by providing for feedback loops of macrophage activation. Being a usual cargo of macrophage- or tumor cell-derived exosomes ncRNAs provide an important mechanism of tumor-stromal cell interaction that contributes significantly to the pathogenesis of various types of tumors. Despite the volume of ongoing research there are still many gaps that must be filled before the practical use of ncRNAs will be possible. In this review we discuss the role of regulatory RNAs in the development of macrophage phenotype. Further we review recent studies supporting the hypothesis that macrophages may affect the properties of tumor cells and vice versa tumor cells influence macrophage phenotype by miRNA and lncRNA transported between these cells by exosomes. We suggest that this mechanism of tumor cell – macrophage interaction is highly promising for the development of novel diagnostic and therapeutic strategies, though many problems are still to be solved.

## Introduction

Macrophages are a heterogeneous cell population consisting of cells of various phenotypes. Within the continuum of macrophage functional states two extremes are designated as classically activated M1 macrophages and alternatively activated M2 macrophages. M1 is characterized by the production of signaling molecules that promote inflammation - TNFα, IL-1beta and others (1). M2 macrophages are characterized by the production of anti-inflammatory cytokines - TGFβ, IL-10 and some others (2). However, the macrophage dichotomy is rather conditional and macrophage phenotype is highly flexible and can be regulated by various factors.

The molecular basis of macrophage polarization by cytokines is quite well understood. The IRF/STAT signaling pathway activated by IFNγ, and various bacterial products *via* TLRs, leads to the development of M1 polarization, while M2 polarization is induced by IL-4 or IL-13. These processes are reversible both *in vitro* and *in vivo* (3, 4). Interferons and TLRs activate the IRF/STAT cascade through STAT1, and M2-stimulating cytokines through STAT6 (2). Additional cytokines or hormones influence the macrophage phenotype in their specific ways. Physical factors, such as hypoxia, may also influence the macrophage phenotype (5).

In the development of solid tumors, macrophages of different phenotypes can play opposite roles. Thus, pro-inflammatory M1 macrophages can suppress tumor progression, while immunosuppressive M2 stimulate angiogenesis and invasion (4, 6, 7). The M1/M2 ratio of tumor associated macrophage population changes significantly with tumor development and depends on the disease stage. For the early stages, M1 macrophages are the predominant population, with tumor development the ratio shifts towards M2 (7, 8). M1 macrophages are able to prevent tumor development, largely due to the presentation of antigens on their surface and the recruitment of CD8+ T cells and NK cells (9). Although interaction of tumor cells and tumor associated macrophages (TAMs) is usually studied in regard of cytokines and other secreted mediators produced by both types of cells, there are several emerging directions of research including regulatory RNA molecules.

## microRNA in defining macrophage phenotype

In addition to cytokines, microRNA plays an important role in macrophage polarization and the performance of the corresponding functions by these cells. MicroRNA is a sequence of ~22 ribonucleotides, their main function is the inhibition of mRNA translation. About 60% of all eukaryotic cell mRNAs contain miRNA complementarity sites, both at the 5’- and 3’-non-coding regions (10). Pre-miRNAs are assembled into a RISC complex, which also includes the RNA-specific endonuclease Dicer and Drosha, which are involved in the processing of pre-miRNA into a mature form, as well as proteins from the Argonaut family (11). Guided by miRNA, the RISC complex is involved in the inhibition of translation of an mRNA (10, 12, 13). RISC can inhibit assembly of the 80S translational complex. The Ago2 protein in the RISC complex competes with the 5’ recognition site of the eukaryotic initiation factor 4G (eIF4G). According to other data, translation inhibition is associated with the interaction of RISC with the anti-associating factor eIF6, which also prevents the assembly of the 80S translation complex (10).

MicroRNAs can be encoded within introns, exons, and between different genes (14). The expression of miRNA is under the control of various transcription factors, but may also depend on the level of already expressed miRNA by the feedback principle with its own transcription factors (11). MicroRNAs can be used as a diagnostic markers for various diseases (11).

A number of miRNAs control the macrophage phenotype and function. Here we provide just several examples of those. In the case of increased expression miR-720 inhibits GATA3 protein, an important regulator of the M2 polarization of macrophages, suppresses the manifestation of the M2 phenotype and shifts it towards M1, and reduces the phagocytic activity of tumor-associated macrophages. Normally, the expression of this miRNA is significantly reduced in M2 macrophages in comparison with M0 and practically does not change when the M1 phenotype is induced. At the same time, stimulation of GATA3 expression in macrophages overexpressing miR-720 contributed to the restoration of the M2 phenotype, which indicates a close relationship between this microRNA and the macrophage phenotype (15).

Another interesting example is miR-127 that was shown to inhibit the B-cell lymphoma receptor Bcl6 and Dusp1 phosphatase, which promotes JNK activation and development of M1 macrophages. The authors demonstrated that overexpression of miR-127 in macrophages significantly increases the expression of pro-inflammatory markers such as IL-6, IL-1β, tumor necrosis factor alpha, and inducible NO synthase (iNOs), typical for M1 polarization (16).

Both miR-720 and miR-127 are expressed in macrophage upon their stimulation with pro-inflammatory stimuli (15, 16), so they can be considered a part of the intracellular machinery, necessary for the macrophage phenotype development. This contribution can be modulated by transfecting macrophages with corresponding miRNA inhibitors.

Various miRNA-mediated patterns have also been shown to be associated with M2 polarization (17). For instance, the miR-23a/27a/24-2 are overexpressed upon macrophage stimulation with M2-associated cytokines and down regulated by M1-associated stimuli. At the same time forced expression of these miRNAs led to M1 phenotype development *via* different mechanisms. Amplification of miR-23a expression enhances activation of the NF-κB pathway by binding to one of the NF-κB suppressors A20 and simultaneously stimulates the expression of M1 cytokines (18). Therefore, these miRNAs can be considered as a part of a negative feedback loop of M2 phenotype development.

MiR-301a was demonstrated to attenuate macrophage migration and phagocytosis in a mouse KO model. This study was done without induction of any specific macrophage phenotype demonstrating that miRNA affects the basic function of macrophages (19).

There are more studies of miRNAs involved in modulation of macrophage phenotype, reviewed elsewhere (20, 21) though our knowledge of the biological significance of observed effects remains limited due to the absence of unified experimental systems (**Table 1**). One of the common shortcomings of many studies on microRNA role in macrophage activation is the absence of time course experiments. Especially important this can be for the induction of M1 phenotype that is in many cases a very rapid event.

**Table 1:** ncRNA in macrophage polarization and TAM-tumor cells interaction.

ncRNA	Source	Effect	Experimental system	Reference
miR-720	macrophages, upon inflammatory stimulation	M1 polarization	Human cell lines	(15)
miR-127	macrophages upon stimulation	M1 polarization	Mouse cell lines	(16)
miR-23a/27a/24-2	macrophages upon IL-4 stimulation	M1 polarization	Mouse cell lines, mouse BMDM	(18)
miR-301a	macrophages	decrease of migration and phagocytosis	Mouse cell lines, mouse BMDM	(19)
lncRNA MM2P	macrophages upon IL-4 stimulation	M2 polarization	Mouse cell lines, mouse BMDM	(22)
lncRNA RPPH1	CRC cells exosomes	M2 polarization	Human peripheral blood monocytes	(23)
miR-155, miR-181, miR-451	M1 macrophages	M1 polarization	Mouse BMDM	(24)
miR-146a, miR-125a, miR-145-5p	M2 macrophages	M2 polarization	Mouse BMDM	(24)
miR-511-3p	M2 macrophages	M2 polarization	Mouse BMDM, mouse TAMs	(25)
miR-193a-5p	TAMs	Renal cell carcinoma progression	Human cell lines	(26)
miR-501-3p	TAMs	Pancreatic cancer progression	Human cell lines	(27)
miR-223	TAMs	Breast cancer progression	Human peripheral blood monocytes derived macrophages	(28)
miR-155-5p and miR-21-5p	TAMs	Colorectal cancer progression	Human TAMs	(29)
miR-181a	Tumor-associated fibroblasts educated TAMs	Breast cancer progression	Human peripheral blood monocytes derived macrophages, human cell lines	(30)

## Long noncoding RNA

In addition to miRNAs, long noncoding RNAs (lncRNAs) can also be involved in macrophage phenotype development (31). Long non-coding RNAs are sequences of more than 200 nucleotides and are not used as templates for protein synthesis, while carrying exclusively regulatory functions (32). It is noteworthy that various tumors are characterized by impaired expression of lncRNAs associated with tumor progression (33, 34). As miRNAs macrophage phenotype modulating lncRNAs can be expressed by macrophages themselves or delivered to macrophages by exosomes or artificial delivery systems.

For instance, MM2P lncRNA is overexpressed in macrophages upon their stimulation with IL-4 and suppressed by LPS stimulation. Further it was demonstrated that transfection of macrophages with MM2P lncRNA enhance M2 polarization of macrophages induced by IL-4 or IL13 (22). The authors also established that MM2P knockdown leads to a decrease in the concentration of phosphorylated STAT6 in macrophages and by this way prevent their M2 polarization (22).

Not only the lncRNAs are expressed in macrophages upon their stimulation with pro- or anti-inflammatory stimuli. LncRNA RPPH1 is expressed in colorectal cancer (CRC) cells and may be transported to macrophages inside exosomes. In macrophages lncRNA RPPH1 triggers M2 development contributing to tumor aggressiveness (23).

## Exosomes

Transfer of molecules by extracellular vesicles (EVs) has been studied as a mechanism of intercellular communication since about 2 decades. EVs is a group of membrane-enclosed vesicles that are naturally released by almost all cell types. EVs are the most important carriers that transport “cargo” from parent cells to target cells, regulating physiological or pathological processes in recipient cells. By origin and size, EVs were originally divided into exosomes (30–200 nm), microvesicles (200–1000 nm), and apoptotic bodies (1–5 μm), but not so long ago, with increasing interest in Other EV subpopulations have also been identified, such as exomers (<50 nm) and large oncosomes (1–10 µm) (35). It has been shown that exosomes carry complex and highly cell-specific cargoes, including DNA, RNA, lipids, metabolites, cytosolic and surface proteins (36). They can be selectively captured by neighboring cells, or cells far from the place of release, and reprogram recipient cells with the help of biologically active molecules contained inside. It is generally accepted that their content can vary greatly depending on the types of cells, their secretion and their current physiological state. Thus, exosomes represent a mechanism of intercellular communication that plays an important role in many cellular processes, including the immune response (37, 38). Exosomes and the molecules they contain may be of prognostic value in chronic inflammation, cardiovascular and renal diseases, lipid metabolism disorders, and cancer (39, 40). Thus, through exosomes, tumor cells influence their microenvironment, which leads to adaptation of the tumor stroma with subsequent stimulation of tumor growth. On the other hand, exosomes secreted by cells of the tumor microenvironment, in particular tumor-associated macrophages (TAMs), may affect tumor growth.

## TAM exosomes

The functions of macrophage exosomes have been widely studied, and the data obtained indicate their key role in disease progression. It should be noted that in recent studies, macrophage extracellular vesicles (EV) are considered to be one of the most important mediators of inflammatory diseases and cancer. As well macrophage EV are thought to be mediators of a positive effect on immunoregulation, tumor therapy, protection against infections, and tissue repair (41).

The content of macrophage exosomes may differ depending on the macrophage phenotype or the composition of their microenvironment. Since macrophages can form a complex mixed phenotype in various diseases or even at different stages of the same disease *in vivo*, it is quite difficult to identify the composition of their exosomes. Proteome analysis revealed different proteins, including cathepsins, 20S proteasome subunits, ribosomal proteins, and heterogeneous nuclear ribonucleoproteins in exosomes released from TAMs, indicating that macrophages may release exosome proteins with increased proteolytic activity and reduced RNA binding capacity (42).

Among the most important molecules contained in macrophage exosomes are various types of RNA molecules. Being protected from ribonuclease degradation within exosomes, ncRNAs can be secreted into various body fluids. MicroRNAs appear to be the most abundant regulatory RNAs in exosomes. In a study by Zhang et al. 109 microRNAs were identified that are differentially expressed in M1- and M2-polarized human and mouse macrophages, including miR-155, miR-181, miR-451 in M1 macrophages and miR-146a, miR-125a, miR-145-5p in M2 macrophages (24). Several miRNAs, miR-146 and miR-155, affect the activation of pathways associated with immune control and the consequences of inflammation (43). Other miRNAs highly expressed in M2 macrophages are miR-511-3p, miR-223 and let-7c, all of which promote M2 polarization (20). MiR-511-3p, which is highly expressed in TAM, targets ROCK2 (Rho-associated helical coil containing protein kinase 2) and maintains the expression of genes associated with M2 polarization (25). TAM-secreted exosomes downregulate TIMP2 expression in RCC cells, promoting vasculogenic mimicry and invasion by miR-193a-5p transfer, which ultimately promotes metastasis (26).

miR-501-3p miRNA isolated from exosomes secreted by tumor-associated M2 macrophages promotes tumor growth and progression of pancreatic cancer. This microRNA inhibits the expression of the TGFBR3 gene, which is an important tumor suppressor, which stimulates an increase in the rate of cell migration and metastasis (27). A decrease in TGFBR3 expression is observed in a number of tumors, which indicates the importance of this cascade in the context of tumor development (44, 45).

The transmission of various microRNAs from macrophages to tumor cells was demonstrated in a study by Mei Yang et al. IL-4 polarized M2 macrophages secrete exosomes containing miR-223. As a result of cocultivation of macrophages with breast cancer cells, it was possible to detect the appearance of this miRNA in tumor cells (28). Data on the differential expression of miR-223 in normal and tumor cells indicate that this miRNA can contribute to the progression of tumors of various types, including renal cell carcinoma and bladder cancer (46, 47).

M2 macrophages are also able to stimulate tumor invasion and angiogenesis through exosomal miRNAs. According to a study by Jingqin Lan et al., in the case of colorectal carcinoma, miR-155-5p and miR-21-5p are transported from M2 macrophages to tumor cells *via* exosomes. In turn, the target of these miRNAs is the BRG1 sequence: this gene is recognized as one of the important suppressors of metastasis in colorectal carcinoma. When miR-155-5p or miR-21-5p interact, a significant drop in the level of BRG1 expression is observed, which may be associated with an acceleration of tumor progression and invasion (29).

Not only tumor cells can modulate TAM phenotype in a way that these cells produce miRNA supporting tumor growth. It was demonstrated that cancer associated fibroblasts stimulate TAMs to express high levels of miR-181a. These TAMs produce miR-181 containing exosomes that activate AKT signaling in breast cancer cells and increase the aggressiveness of the tumor (30).

Recent studies have shown that TAM exosomes also contain various long non-coding RNAs (lncRNAs). The interactions of lncRNA with RNA, DNA, and proteins allow them to regulate gene expression at several levels, so roles in gene regulation are usually divided into epigenetic, transcriptional, and post-transcriptional levels. LncRNAs reside either in the cytoplasm or in the nucleus, where they can interact with miRNAs, mRNAs, RNA-binding proteins (RBPs), transcription factors, and chromatins and act as enhancer-like RNAs (48). Accumulated data have shown that cytoplasmic lncRNAs can be involved in gene regulation at the post-transcriptional level, including acting as ceRNAs and protecting target mRNAs from repression (49).

Accumulated data show that lncRNAs are actively involved in the regulation of many fundamental biological processes of development. At the moment, their participation in epigenetic regulation (gene dosage compensation, genomic imprinting), cell differentiation, and organogenesis has already been shown (50). Some lncRNAs—MALAT1, HOTAIR, and ANRIL—are associated with various pathologies, including cancer (51). Extracellular vesicular transmission of myeloid-derived HIF-1α-stabilizing long non-coding RNA (HISLA) is positively correlated with poor overall survival in breast cancer patients. It has also been shown that HISLA within TAM-derived exosomes can promote aerobic glycolysis, apoptosis resistance, and chemoresistance in breast cancer cells (52).

## Tumor cell-derived exosomes

Studies have shown that tumor cells produce much more exosomes than normal cells. Due to the presence of adhesion receptors and ligands specific for various types of cells and tissues on their membranes, these exosomes “target” certain types of cells, delivering the widest spectrum of biological molecules. Exosomes secreted by tumor cells carry various proinflammatory and immunosuppressive factors, such as macrophage migration inhibitory factor (MIF) and PD-L1, which act in nearby or distant tissues or organs to induce vascular permeability, inflammatory infiltration, extracellular matrix remodeling, and downregulation. immune response. Activated stromal cells can release a variety of cytokines and chemoattractants *via* exosomes, such as IL-6, IL-8 and S100A9, which promote tumor cell proliferation and invasion, as well as the acquisition of chemoresistance and stem cell phenotype (53).

In addition to proteins, miRNAs contained in tumor cell-derived exosomes may affect macrophage polarization. Exosomal miR-301a-3p stimulates macrophage polarization to the M2 phenotype *via* the PTEN/PI3Kγ signaling pathway. Circulating miR-301a-3p levels are positively correlated with later tumor stage, TNM grade; an increase in the level of circulating this microRNA is associated with a worse prognosis of survival in case of pancreatic cancer (54). Tumor cell derived exosomal miR-138-5p inhibits KDM3B expression, thereby promoting the M2 phenotype and blocking M1 polarization. In the case of breast cancer, an increase in the content of exosomal miR-138-5p was associated with a worse prognosis (55). Considering the plasticity of macrophage phenotype it would be important to investigate the stability of macrophage phenotype change induced by tumor cell-derived miRNA.

## Conclusions

The investigation of exosome-mediated intercellular communication between tumor cells and tumor-associated macrophages (TAMs) has provided valuable insights into the potential for identifying new targets for anticancer therapy, particularly regulatory RNAs. The results of this research suggest that the inhibitory effects mediated by M1-like macrophages can be a promising approach for cancer therapy. Macrophage reprogramming towards the M1 phenotype, through the modification of exosomal cargoes, may serve as a strategy for suppressing tumor growth.

However, despite the extensive research in this field, there are still many gaps in our understanding of the complex exosome-mediated communication process between tumor cells and macrophages. One of the main challenges is the lack of comparability between different experimental systems, particularly with regards to non-coding RNAs in macrophages and the limited comparability between mouse and human macrophage cell lines (**Table 1**). Additionally, there is a need for a more nuanced approach to the selection of macrophage phenotype markers, rather than relying solely on the M1/M2 dichotomy. Further research in this area should consider the dynamic changes in macrophage phenotype that can occur in response to different stimuli, and the use of multiple markers to accurately characterize macrophage phenotype.

In order to fully understand the impact of non-coding RNAs on macrophages and tumor cells, extensive kinetics studies are crucial. Studies have been performed to assess the kinetics of LPS-induced TNF production (56) and the cytokine production induced by IFN-γ or IL-4 in macrophages (57), revealing the complexity of macrophage behavior over time. Similar studies of ncRNAs can provide insight into the regulatory networks controlling macrophage biology and identify key hubs that can be targeted for therapeutic intervention. Additionally, further investigation into the role of exosomes in tumor progression and the cross-talk between different cell types within the tumor microenvironment will provide a more comprehensive understanding of the complex interplay between tumor cells, macrophages, and the surrounding microenvironment. This knowledge can be leveraged to design more effective, targeted therapeutic strategies for cancer treatment.

## Author contributions

OK wrote the manuscript. MS wrote the manuscript. AE wrote the manuscript. AP wrote and proofread the manuscript. KS wrote and proofread the manuscript. AG designed the concept, proofread the manuscipt. All authors contributed to the article and approved the submitted version.
